# Edible insects – defining knowledge gaps in biological and ethical considerations of entomophagy

**DOI:** 10.1080/10408398.2018.1468731

**Published:** 2018-05-17

**Authors:** Isabella Pali-Schöll, Regina Binder, Yves Moens, Friedrich Polesny, Susana Monsó

**Affiliations:** a Comparative Medicine, Messerli Research Institute of the University of Veterinary Medicine Vienna, Medical University Vienna and University Vienna, Vienna, Austria; b Institute of Animal Husbandry and Animal Welfare, Department of Farm Animals and Veterinary Public Health, University of Veterinary Medicine Vienna, Vienna, Austria; c Anaesthesiology and Perioperative Intensive Care, University of Veterinary Medicine, Vienna, Austria; d AGES Academy, Austrian Agency for Health and Food Safety (AGES), Vienna, Austria; e Ethics and Human-Animal Studies, Messerli Research Institute of the University of Veterinary Medicine Vienna, Medical University Vienna and University Vienna, Vienna, Austria; f Section of Moral and Political Philosophy, Institute of Philosophy, Karl-Franzens-Universität Graz, Graz, Austria

**Keywords:** Edible insects, ethical aspects, legal situation, novel food, nociception, rearing, allergenicity, sentience, entomophagy

## Abstract

While seeking novel food sources to feed the increasing population of the globe, several alternatives have been discussed, including algae, fungi or *in vitro* meat. The increasingly propagated usage of farmed insects for human nutrition raises issues regarding food safety, consumer information and animal protection. In line with law, insects like any other animals must not be reared or manipulated in a way that inflicts unnecessary pain, distress or harm on them. Currently, there is a great need for research in the area of insect welfare, especially regarding species-specific needs, health, farming systems and humane methods of killing. Recent results from neurophysiological, neuroanatomical and behavioral sciences prompt caution when denying consciousness and therefore the likelihood of presence of pain and suffering or something closely related to it to insects. From an animal protection point of view, these issues should be satisfyingly solved before propagating and establishing intensive husbandry systems for insects as a new type of mini-livestock factory farming.

## Abbreviations

APA: Austrian Animal Protection Act
EC: European Community
EFSA: European Food Safety Agency
EU: European Union
FDA: Food and Drug Administration
OJ: Official Journal of the European Union
PAP: processed animal protein

## 1. Introduction

Anthropo-entomophagy, the consumption of insects as food by humans, is no novel phenomenon, because obviously it has been practiced since the very early development of human beings (Sponheimer et al. 2005). Humans initially had an insectivorous diet, and only with subsequent evolution came fruits, vegetables and meat, when humans began hunting and eating other mammals (reviewed in Ramos-Elorduy 2009). Today, entomophagy is still a form of nutrition in over 100 countries, mainly in Africa, Asia and Latin America, with over 2300 insect species used for consumption by about 3000 ethnic groups (van Huis et al. 2013). When seeking novel food sources to feed the increasing population of the globe, several alternatives have been discussed, including algae, fungi or *in vitro* meat. In addition, there are several arguments on the table for promoting insects as novel food and novel feed worldwide, including in industrialized Western countries.

First, there is the nutritional value of insects, namely their high content of biologically valuable protein, optimal fatty acids and several favorable micronutrients like copper, iron, magnesium, riboflavin, biotin and others (Rumpold and Schluter 2013).

Further reasons for consuming insects are environmental. De Goede et al. summarize the presumed environmental advantages of insect farming (De Goede, Kapsomenou, and Peters 2013), although one has to admit that —to the best of the authors' knowledge— there is no reliable calculation of the overall ecological- or water-footprint or the risks of large-scale mono-cultured insect farms in Western climate conditions. However, another advantage seems to be their capacity for bioconversion, as insects can be reared on residual waste, such as manure of poultry, swine and cow, as well as fish waste (Ramos-Elorduy 2009; Rumpold and Schluter 2013; Ocio, Vinaras, and Rey 1979). This feed can be converted very efficiently into biomass with a relatively low energy input, due to the poikilothermic metabolism of insects (van Huis et al. 2013). The rearing *per se* could be realized with much less land use than other livestock (Oonincx et al. 2010), and with only low space requirement, by piling multiple stories on top of each other. Furthermore, it seems that emissions of greenhouse gases and ammonia of commercially-reared insects are lower than in the case of conventional livestock (Oonincx et al. 2010).

As there is a large phylogenetic distance between insects and humans and other mammals, the risks of transmission of diseases from insects to humans are most probably very low (van Huis et al. 2013). Finally, the introduction of insects as feed, e.g. as substitute for fishmeal, protects other species and thereby biodiversity (Rumpold and Schluter 2013). All in all, these arguments suggest many advantages of including edible insects as novel food on our plates and novel feed in the bowls. However, in addition, a number of controversial aspects have to be considered, such as food and feed safety, consumer information, and animal protection issues brought about by the large scale breeding, rearing and killing of insects.

## 2. Legal situation – the European perspective

The usage of insects for human purposes (e.g. production of silk and honey) has a long tradition in different parts of the world. The same is true for entomophagy, i.e. the use of insects for human consumption. In Western societies, however, the use of insects as food is a very small niche market so far (EFSA Scientific Committee 2015), although over the last years interest in using insects as food and feed has been increasing and propagated for various reasons (van Huis et al. 2013; EFSA Scientific Committee 2015).

From the legal perspective, large-scale farming of insects for the production of food and feed (for other farm animals) raises primarily two issues: (i) the question of food safety, and (ii) the issue of animal protection.

### 2.1. Food safety

With respect to the food safety issues involved in the use of insects as food and feed, legislators have to pay special attention to the general guidelines that apply to novel food, as well as to any potential allergenicity risks.

#### 2.1.1. Regulation of novel food

According to Regulation (EC) No 1069/2009^1^ 1Regulation (EC) No 1069/2009 of the European Parliment and of the Council of 21 October 2009 laying down health rules as regards animal by-products and derived products not intended for human consumption and repealing Regulation (EC) No 1774/2002 (Animal by-products Regulation). *OJ L 300/1, 14/11/2009*., insects are regarded as “farmed animals”. Insects, either whole or processed, are food as defined by Art. 2 of Regulation (EC) No 178/2002^2^ 2Regulation (EC) No 178/2002 of the European Parliament and of the Council of 28 January 2002 laying down the general principles and requirements of food law, establishing the European Food Safety Authority and laying down procedures in matters of food safety, *OJ L 031, 01/02/2002*. and, like any other animals intended for human ingestion, are subject to the supranational and national legislation on food safety^3^ 3Cf. esp. Regulation (EC) No 852/2004 of the European Parliament and of the Council of 29 April 2004 on the hygiene of foodstuffs, *OJ* *L 139/1, 30/4/2004*; Österr. Bundesgesetz über Sicherheitsanforderungen und weitere Anforderungen an Lebensmittel, Gebrauchsgegenstände und kosmetische Mittel zum Schutz der Verbraucherinnen und Verbraucher (Lebensmittelsicherheits- und Verbraucherschutzgesetz – LMSVG), BGBl. I Nr. 13/2006 in its current version.. Generally speaking, the processing and storage of insects and products of insect origin have to follow the same health and sanitation regulations as conventional foodstuff.

Additionally, the regulatory framework for novel food is applicable. According to EU-legislation, novel food is defined as foods and food ingredients that have not been consumed to a significant degree by humans in the EU prior to May 15th, 1997, when the first supranational regulation on novel food came into force^4^ 4Regulation (EC) No 258/97 of the European Parliament and of the Council of 27 January 1997 concerning novel foods and novel food ingredients, *OJ L 043, 14/02/1997*..

Novel food in general and insects in particular may be connected with a series of potential risks for human health (e.g. biological and chemical hazards, allergenicity) and the environment, which have to be assessed and minimized by regulatory requirements. Because there is little experience with the production and consumption of these foods, they have to undergo a standardized safety assessment process before being allowed to be marketed in the EU, in order to make sure that they do not represent a risk to the consumer. The first EU regulation on novel food, dating back to 1997, was superseded by a new European Novel Food Regulation, which came into force on December 31st, 2015 and has been legally binding for the member states since January 1st, 2018^5^ 5Regulation (EU) 2015/2283 of the European Parliament and of the Council of 25 November 2015 on novel foods, amending Regulation (EU) No 1169/2011 of the European Parliament and of the Council and repealing Regulation (EC) No 258/97 of the European Parliament and of the Council and Commission Regulation (EC) No 1852/2001, *OJ L 327/1, 11/12/2015*.. This new regulatory framework is primarily concerned with centralizing, simplifying and expediting evaluation and authorization procedures. Whereas applications for authorization of a novel food item were previously assessed in the individual member states, they now are evaluated centrally by the European Food Safety Authority (EFSA). Basically, individual authorizations are replaced by general (generic) authorizations, applying not only to the applicant, but to all distributors of the relevant foodstuff.

With regard to foods that have been traditionally consumed in third-party countries, safety evaluation will be more efficient in order to make access to the EU market easier. In this way, according to Art. 14 of the new Novel Food Regulation, a simplified authorization procedure (notification) is sufficient if the safe use of the relevant food outside the EU can be demonstrated for a period of at least 25 years and no appeal is lodged by the member states or the EFSA; in the latter case, the relevant food item is subject to the standard authorization process.

The use of insects as feed currently is limited to pets (such as birds, reptiles and amphibians), because of the feed ban provisions of Regulation (EC) No 999/2001^6^ 6Regulation (EC) No 999/2001 of the European Parliament and of the Council of 22 May 2001 laying down rules for the prevention, control and eradication of certain transmissible spongiform encephalopathies, OJ *L 147, 31/05/2001*. (TSE Regulation), which do not allow insect Processed Animal Protein (PAP) to be fed to farmed animals due to lack of a safety profile (EFSA Scientific Committee 2015).

#### 2.1.2. Allergenicity

One of the safety points that have to be considered according to the Novel Food directive is the allergenicity of edible insects. For many insect species, it is already well-established that long-term, high-antigen environmental exposure, e.g. of professional insect farmers, leads to respiratory sensitization in a percentage of up to 50–60% of individuals rearing grasshoppers (Pener 2014; Lopata et al. 2005) or silkworm (Uragoda and Wijekoon 1991). Allergenicity also has to be considered in the domestic context. A rather new development is the acquisition of reptiles as pets in homes, which are fed live grasshoppers, and here the owner has to consider the sensitization potential via the lung or skin (Jensen-Jarolim et al. 2015).

Regarding the consumption of insects, there has been some anecdotal data from China, which report 54 cases of anaphylaxis due to consumption of short- or long-horned grasshoppers between 1980 and 2007 (Ji et al. 2009). Furthermore, around 1000 anaphylaxis cases per year occur due to consumption of silkworm in China (Ji et al. 2008). In recent studies from the Western countries, the potential for cross-reactivity of crustacean- or house dust mite-allergic patients to mealworm or grasshopper consumption has been shown (Verhoeckx et al. 2016; Broekman et al. 2016; Broekman et al. 2017; Broekman et al. 2017; Broekman et al. 2015; Verhoeckx et al. 2014; van Broekhoven et al. 2016). Accordingly, the guidelines of the Austrian Federal Ministry of Health and Women's Affairs for insects strongly recommend the labeling of edible insects and products thereof with the potential risk of allergic reactions in people who are allergic to crustaceans or house dust mites^7^ 7Leitlinie für gezüchtete Insekten als Lebensmittel. https://www.verbrauchergesundheit.gv.at/lebensmittel/buch/codex/beschluesse/Insekten_LL.pdf?5th0ev.

Importantly, also primary sensitization can take place when mealworms are eaten by humans (Broekman et al. 2017). These studies furthermore point towards individual sensitization, meaning that having an allergic reaction to one insect species does not necessarily mean that an allergic reaction to all insects will take place (Broekman et al. 2017). Therefore, as there are a number of different insect species around and they might contain different proteins, the allergenic potential probably needs to be evaluated individually.

Taken together, neither the knowledge about cross-reactivity nor about primary sensitization regarding edible insects is so far complete. Furthermore, as insects are unlikely to be consumed in a raw or whole state in our countries and therefore presumably will arrive at the market in a processed form, it would be ideal to reveal and legislate the most efficient treatment and technological processes to reduce the primary sensitization capacity as well as cross-reactivity, which could be successfully performed for migratory locust by protein hydrolysis (own unpublished data).

Apart from labeling potential allergenic reactions or cross-reactivities, novel food must not be misleadingly labeled, which is especially important with regard to products made of or containing ingredients of insect origin that are not recognizable as insects (e.g. powder, granules, paste).

### 2.2. Animal Protection

Large-scale farming of insects (mini-livestock) may cause a variety of animal protection issues, especially in the areas of rearing, husbandry and killing, which seem to have been widely neglected by regulatory frameworks and recommendations so far.

Collecting insects in the field would make for a rather uncertain source of food. If insects shall play an important role in human nutrition, efficient mass-rearing with minimized input of materials, energy and labor is needed. Bee-keeping for producing honey and breeding silk moths on mulberry for silk production have a history of some thousands of years. For decades there has been a lot of experience in mass-rearing of different insect plant pests to use either directly in plant protection (sterile insect technique) or as feed for mass rearing of beneficials.

**Figure 1.**
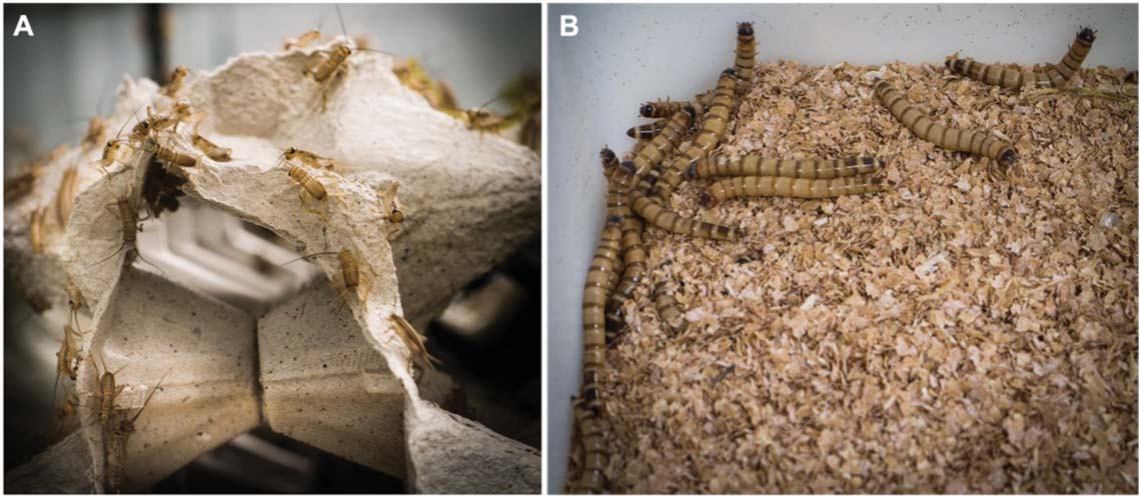
Crickets *Acheta domesticus* (A) and Yellow mealworm *Tenebrio molitor* (B) reared as feed for reptiles and birds in the zoo.

There is a lot of practical experience in breeding insects as feed for animals in zoos (Figure 1) or private terrariums. Most of these species are of interest for human nutrition, too.

The insect species eligible for mass-rearing for human nutrition (De Goede, Kapsomenou, and Peters 2013; Schneider 2009; Erens et al. 2012) will be those that allow for fast growth under easy-to-maintain climatic conditions, as well as cheap and disposable feed. In order to ensure minimum welfare standards, the species in question should also have a social behavior that allows the individuals to be kept in relatively high densities without cannibalism. These demands are met by several insect species:

• Species with provenience in tropic or subtropic regions with rather stable climatic conditions (e.g. many locusts)
• Synanthropic species (e.g. cockroaches)
• Stored-food pests (e.g. mealworm, *Tenebrio molitor L*.)
• Saprophagous or carrion feeding species (e.g. Black soldier fly, *Hermetia illucens L*.)

Predatory insets will most probably not be suitable for mass-breeding for human nutrition, because this would require a mass-breeding of living insect feed in an upstream mode and raise costs and necessary input.

For a stable mass-rearing of insects, a high level of hygiene is a basic requirement. Otherwise the breeding will collapse because of viral, bacterial or fungal pollution (Maciel-Vergara and Ros 2017). Hygiene requirements for human food have specialized standards, and the same will apply to the rearing of insects from egg to adult.

The collection of the growth stage that is envisaged for usage or processing for consumption, e.g. larvae or adult insects, and the way to kill the specimen are both especially challenging. There is a need for a fast and non-poisonous as well as non-hurting killing of the target growth stage of the specimen before it is prepared for food or feed use, as one method for killing might not be optimal for every growth stage of a species. Fast freezing and/or treating with boiling hot water are the two preferred ways, where the most “humane” way needs to be assessed individually for every species and growth stage, e.g. fast freezing seemed to be better for mealworm than boiling (Adámková et al. 2017).

Although according to the Scientific Opinion rendered by the ESFA in 2015, the “general animal (vertebrate) health and welfare regulations should also apply for insects”, the Art. 1 no. 2 d) of Council Directive 98/58/EC concerning the protection of animals kept for farming purposes^8^ 8Directive 98/58/EC of 20 July 1998 concerning the protection of animals kept for farming purposes, OJ *L 221, 08/08/1998*. explicitly excludes “any invertebrate animal” from its scope. Furthermore, the EFSA itself defined health and welfare of insects as an issue outside the terms of reference of the above mentioned document. Similarly, a guideline on farmed insects used as food, published by the Austrian Federal Ministry of Health and Women's Affairs, which is competent for food safety as well as for animal welfare, does not even touch upon the issue of animal protection,^9^ 9Ministry of Health and Women's Affairs (now Ministry of Health) (2017): Guideline for insects bred as food. Published with reference no: BMGF-75210/0003-II/B/13/2017, 15/02/2017. although the Austrian Animal Protection Act^10^ 10Federal Act on the Protection of Animals (Animal Protection Act – APA; *Tierschutzgesetz - TSchG*), Federal Law Gazette I no 118/2004, Art. 2, in its current version. (APA) partly covers all invertebrate animals and thus also applies to insects.

Although so far there is no conclusive evidence of sentience in insects, some entomologists proposed to apply humane killing techniques as early as in the first third of the 19th century (Cooper 2011). In 1980, entomologist V.B. Wigglesworth recommended anesthetizing insects prior to traumatizing manipulations in laboratory settings, although he himself was convinced that insects by and large do not feel pain (Eisemann et al. 1984). This precaution, according to Wigglesworth, “not only facilitates handling, but also guards against the remaining possibility of pain infliction and, equally important, helps to preserve in the experimenter an appropriately respectful attitude towards living organisms whose physiology, though different, and perhaps simpler than our own, is as yet far from being completely understood” (Wigglesworth 1980). Thus, in laboratory settings it is increasingly regarded as unacceptable to conduct invasive procedures on invertebrates without “some form of chemical restraint, preferable through the use of an agent with known anesthetic properties” (Cooper 2011; Cooper 2001). Interestingly, despite this progressive attitude towards insects' possible sentience, invertebrates (except cephalopods) are not subject to contemporary animal experimentation legislation of the EU^11^ 11Cf. Art. 1 no 3 of Directive 2010/63/EU of the European Parliament and of the Council of 22 September 2010 on the protection of animals used for scientific purposes, *OJ L 276/33, 20/10/2010*; § 1/1 Austrian Federal Act on Animal Experiments (*Tierversuchsgesetz* 2012 – TVG 2012), Federal Law Gazette I no 114/2012, 28/12/2012, Art. 1..

From the legislative point of view it is not necessary that the sentience (i.e. whether they are capable of experiencing pain or suffering) of animals that fall within the scope of animal protection legislation be proven. With regard to its ethical foundation, animal protection law may be divided into two types of clauses: (1) pathocentrically-oriented pieces of legislation, which consider only sentient animals, and (2) biocentric clauses, which pertain to all animals. Although contemporary animal protection legislation is characterized predominately by pathocentrism, being mainly concerned with prohibiting the infliction of unjustified pain and suffering on sentient animals and with safeguarding their well-being, it also includes biocentric elements, which typically are independent of an animal's (actual or proven) sentience (Binder, Grimm, and Schmid 2009).

Thus, the Austrian APA partially includes insects within its scope (§ 3/1 and 2 APA): while those parts of the APA relating to husbandry conditions (§§ 13ff. APA) and the obligation of animals' keepers (§ 12 APA) apply to vertebrates, cephalopods and decapodes only, two important groups of provisions, namely the ban of animal cruelty (§ 5 APA) and the prohibition of killing animals without a justifying reason (§ 6 APA), apply to all invertebrates; the same is true for the general requirements relating to the methods of killing (§ 32 APA), which stipulate that unnecessary pain and suffering must be avoided when killing an animal.

In line with § 5 APA (animal cruelty), insects, like any other animals, must not be reared or manipulated in a way that inflicts unnecessary pain, distress or harm on them. Regarding animals whose sentience has not been proven, “harm” is the most important type of interdicted impairment, because it is commonly agreed upon that the concept of “harm” (“Schaden”) covers any human-induced worsening of an animal's condition (Binder 2014; Kluge 2002; Lorz and Metzger 2007; Hirt, Maisack, and Moritz 2015), irrespective of the animal's subjective perception. Thus, a fly is harmed if one of its legs or wings is torn off, because, despite possible efforts to compensate for the loss, the animal, as perceived from the outer perspective, definitely is worse off than before, even if it is assumed that it neither feels any sort of pain nor is suffering from distress.

The question of whether insects are able to feel pain remains controversial (Elwood 2011) and although it is – as mentioned above – ultimately irrelevant from the legal point of view whether there is any evidence for sentience in insects, it is interesting from the scientific as well as from the ethical point of view that there are indications supporting the possibility of some sort of pain experience in some taxa of insects. These indications, namely, the well-established fact of nociception, the evidence of endogenous opioids in insects and their (at least) rudimentary ability to learn to avoid noxious stimuli (Eisemann et al. 1984; Elwood 2011) are discussed in detail under “Physiological considerations” in the present paper. These indications should be regarded as sufficient to strongly support the precautionary principle, which is inherent in biocentric animal protection legislation, granting the benefit of the doubt to entities that are profoundly different from us and whose potential “inner lives” cannot be conclusively accessed because of epistemological^12^ 12Epistemology is the study of knowledge. An epistemological problem is one related to the methods we use to acquire knowledge. boundaries.

As mentioned above, the APA's regulatory framework includes insects only partially, for § 3/2 APA stipulates that the requirements laid down for animal husbandry do not apply to invertebrates (other than cephalopods and decapods). The large-scale farming of insects may, however, raise a range of welfare issues, if their specific, species-related needs are not considered appropriately. Among other problems, disease and high mortality caused by inappropriate environmental conditions (e.g. temperature, humidity, light), cannibalism due to lack of space or malnutrition, and inhibition of natural behavior, e.g. in migratory species like locusts, are mentioned in the literature (Schneider 2009; Erens et al. 2012; Eisemann et al. 1984). Despite this fact, in Austrian animal protection legislation there are currently no provisions on the rearing and husbandry of insects, neither in the general framework of the APA (which establishes an obligation to consider physiological and ethological needs of animals and to prevent husbandry-related diseases as well as overstraining animals' adaptability), nor with regard to taxa-specific minimum requirements on statutory level.

Another path is taken by the Dutch Animal Act^13^ 13Animal Act 31 389 - Een integraal kader voor regels over gehouden dieren en daaraan gerelateerde onderwerpen (Wet dieren)., which came into effect in 2013 and comprises European Animal Health Law as well as provisions in the area of animal protection. The latter part of the Dutch Animal Act, which is based on the Five Freedoms defined by F.W.R. Brambell in 1965 (Brambell 1965), lists a number of insect species as “production animals”, whose well-being needs to be respected. Thus, according to Dutch legislation, also insects should be free from thirst, hunger and inappropriate feed, physical and physiological inconvenience, pain, injury, and disease, fear and chronic stress, and finally from limitations on natural behavior (De Goede, Kapsomenou, and Peters 2013; Erens et al. 2012).

Commenting on the Dutch legislation, De Goede et al. (De Goede, Kapsomenou, and Peters 2013) make the following suggestions with respect to Brambell's five freedoms (Brambell 1965):

1. Freedom from thirst, hunger and inappropriate feed: Insects can be fed multiple plant and animal sources, including waste (which is an environmental advantage). They tend to get enough water from their feed, but some species are prone to dehydration if there is not enough water provisioning, and low humidity may negatively influence food conversion efficiency (which may mean that it also implies a negative subjective state for the insects).
2. Freedom from physical and psychological inconvenience: Many species are nocturnal, so a place to shelter during the day may be necessary, in addition to the provision of a day/night rhythm.
3. Freedom from pain, injury and disease: With regard to the freedom from pain, this requirement is problematic within the given context because of the lack of consensus on insect pain. It should, however, be kept in mind, that injury as well as disease are also relevant for animals that are actually or presumably insentient, because they invariably harm animals from an objective (external) perspective. When it comes to humane killing methods, very little is known, although freezing is usually considered humane and is currently the main killing technique. And lastly, with respect to the freedom from disease, there is such little scientific knowledge that this freedom cannot currently be provided. When facilities are infected by a virus, there tend to be high mortality rates.
4. Freedom from fear and chronic stress: There is a need to investigate whether current rearing methods cause stress, and whether it can be avoided. However, again, it is unknown whether insects can experience these states subjectively.
5. Freedom from limitations in natural behavior: Insect farms tend to have a higher population density than natural populations, which can lead to overheating. Different stages within the lifecycle may require different rearing conditions. With regard to certain specific insect species, it is quite obvious that husbandry systems would prevent normal behavior. Thus, locusts, for example, are not allowed to perform their extensive migratory and flying behavior in captivity, which could be detrimental for welfare.

Despite this precautionary principle, it could still be preferable to rear insects over traditional livestock as a food source, because even if (some taxa of) insects do experience (some kind of) pain, it does not seem far-fetched to suppose that they are less sentient than vertebrate species, especially mammals. Here it is interesting to note that laboratory animal legislation establishes as mandatory to choose those experimental designs which “involve animals with the lowest capacity to experience pain, suffering, distress or lasting harm.”^14^ 14Art. 13/2/b) Directive 2010/63/EU of the European Parliament and of the Council of 22 September 2010 on the protection of animals used for scientific purposes, *OJ L 276/33, 20/10/2010*; § 6/1/9 Austrian Federal Act on Animal Experiments. This approach, enshrined in laboratory animal legislation since its very beginning in the last third of the 19th century (Binder 2014), might lead to the far-reaching consequence that the usage of insects as food source should have priority over the consumption of traditional livestock for reasons of animal welfare (Principle of Refinement).

## 3. Physiological considerations

The intentional mass killing of insects for reasons of pest control, their incidental killing during daily activities as well as the use of insects for biological research inevitably raise the question of pain perception in these animals (Eisemann et al. 1984). Today, the increasing importance of insects as a commercial source of protein for human consumption further boosts the interest in the topic.

During the last decades, important progress has been made in understanding the mechanisms of pain perception in mammals and key findings are reported in current editions of major textbooks (Tranquilli, Thurmon, and Grimm 2013; Grimm et al. 2015).

In summary, there exists a general agreement that two key components must be present for pain to be experienced: The first component is the ability of an animal to detect and react to noxious stimuli by moving itself or the affected part of its body rapidly away from the stimulus. This phenomenon — “nociception” — may be a pure reflex response, which not necessarily involves pain sensation. For the latter, a second component is necessary, which represents the individual emotional and subjective interpretation of the nociceptive experience — and this requires a component of consciousness.

It has been shown that in vertebrates pain is experienced as a result of central processing of input from free endings of nociceptive nerves, so called nociceptors. Nociceptors detect stimuli that are potentially harmful, like extreme temperatures, irritant chemicals, electrical shocks or pronounced mechanical interference. Most receptor endings are responsive to several types of stimuli.

The signals generated from nociceptors travel via nerve fibers to the spinal cord. At the spinal level, nociceptive input may trigger immediate somatic and sympathetic protective reflexes (nociceptive reflex). At the same time, the signal output is locally processed and transmitted to the brain via ventro-lateral fiber tracts in the spinal cord, resulting in pain sensation. Considerable modulation of the nociceptive input occurs at the spinal level via ascending and descending inhibitory and excitatory pathways, involving various neurotransmitters, substance P and endogenous opioid peptides. At the level of the brain, the thalamus redirects information to different areas in the cerebral cortex for interpretation, whereas the limbic system is responsible for the emotional response. Eventually this can result in non-reflexive responses, like for instance aggression or learned avoidance.

In the early eighties, Eisemann stated on the basis of different arguments that insects are unlikely to experience pain (Eisemann et al. 1984). First, the nervous system of insects differs greatly from that of vertebrate animals, as it has a relatively simple organization and much fewer neurons. They lack the higher neurological structures that translate an aversive stimulus into an emotional experience. In the absence of nociceptors and spinal reflex mechanisms, the aversive responses by insects — which may resemble the nociceptive reflex — are thought to occur following activation of particular nervous system programs activated by excessive or abnormally-patterned non-nociceptive sensory input. In contrast, the discovery of endogenous opioid peptides and their receptor sites in some insects (El-Salhy et al. 1983; Stefano and Scharrer 1981) and the modulation by opiate agonists and antagonists of nociceptive-type responses (Zabala et al. 1984) may indicate — in analogy with their role in mammals — a capacity for pain perception. Opioid peptides are known to also play a role in several other physiological processes and thus their presence *per se* is not a proof of the existence of mechanisms leading to pain perception (Dyakonova 2001). These aforementioned facts, coupled with behavioral observations of injured insects, which e.g. continue normal feeding whilst heavily injured, showed, according to Eisemann, that insects do not have a “pain sub-program” (Eisemann et al. 1984).

Since then, important progress has been made in trying to answer the question of whether insects can experience pain and thus have a form of consciousness. Research from the last decades as reviewed by Elwood (Elwood 2011) allows a more differentiated view on the issue. More is known about the possible neurophysiological pathways in insects confronted with aversive conditions. Several neurotransmitters typically involved in vertebrate pain pathways, such as serotonin and substance P, have been identified in insects (El-Salhy et al. 1983; Wurden and Homberg 1995).

In line with what happens in mammals, hormone-mediated responses to stressful environmental conditions have been identified in some insects. Various kinds of nociceptive neurons responding e.g. to mechanical and thermal stimuli using molecular mechanisms similar to nociceptive signal generation have been identified in the fruit fly *Drosophila melanogaster* (Hwang et al. 2007). In fruit flies, multi-dendrite nociceptive neurons have been identified with ascending projections that cross the midline to innervate contralateral postsynaptic targets (Guo et al. 2014). This bears some similarity with pain processing in vertebrates, but whether the brain of the larvae is involved in perception of the noxious stimulus or whether lower-level processing in the abdominal or thoracic ganglion plays a role remains to be determined. Nowadays, while mammalian animal models are still the most frequently used for the study of acute and chronic pain, the role of the fruit fly as a model for the study of nociception and the screening of potential analgesic substances has gained a lot of interest (Leung et al. 2013; Manev and Dimitrijevic 2005), not in the last place because of restrictive regulatory legislation associated with the use of vertebrate animals in research. The use of the fruit fly has also facilitated research into the genetics of nociception with yields of evidence of evolutionary continuities across vertebrates and invertebrates, e.g. in the role of opioids in protection and survival of organisms.

While the complexity of insect behavior and the apparent involvement of complex processing of neural information is now well recognized, it is still thought that they lack the brain capacity to experience emotion and evaluate injury, which are inseparable from the experience of pain (Gullan and Cranston 2014).

Recently, Barron and Klein reviewed and compared the structure and functions of the vertebrate midbrain with the brain of insects (Barron and Klein 2016). They write that “insect behavior involves multiple layers of filtering of sensory information to support selective attention to stimuli that are salient and suppression of representation of irrelevant stimuli”. The authors therefore presume that structures in the insect brain function similarly to the human midbrain, an area that is essential for subjective experience in humans and where also consciousness is thought to emerge. They claim that consciousness may be very ancient in evolution rather than something that evolved only in “higher” vertebrates.

In conclusion, recent results from neurophysiological, neuroanatomical and behavioral sciences prompt caution when denying consciousness, and therefore the likelihood of presence of pain and suffering or something closely related to it, to insects (Tiffin 2016). This strongly underlines earlier statements (Cooper 2011; Wigglesworth 1980; Elwood 2011; Smith 1991) that while awaiting results of further research one should consider the possibility that at least some insect species might suffer pain and, as a precaution, always ensure humane handling of these animals, including the application of anesthesia and analgesia for painful procedures and humane killing techniques.

## 4. Ethical considerations

### 4.1. The moral importance of sentience

One of the first questions that needs to be asked when discussing insect ethics, and arguably the most important one, is whether the species we are dealing with is *sentient*. Indeed, the answer to this question determines whether or not it makes sense to speak about insect ethics *altogether*. Asking whether insects are sentient is not the same as asking whether they possess nociception. Nociception, as discussed above, can be defined as the “capacity to respond to potentially damaging stimuli” (Adamo 2016). In contrast, sentience is the capacity to *feel*, to undergo *subjective, conscious experiences*. These two capacities are in principle independent of each other. In the case of humans, for instance, “it is possible to have nociception without pain, and pain without any activity in nociceptive fibres” (Adamo 2016; Hardcastle 1997). While it is well established that insects possess a capacity to respond to noxious stimuli (Eisemann et al. 1984; Adamo 2016), theoretically it is possible for these responses to occur without the presence or mediation of any conscious mental states, that is, without sentience.

Asking whether insects are sentient may not be enough, since not all forms of sentience may be relevant for morality. For instance, Birch distinguishes two senses of the term ‘sentient,’ only one of which is considered important for ethics (Birch 2017). In a broad sense, sentience is equal to what is often called ‘phenomenal consciousness.’ If an individual possesses sentience in this sense, it can be said that there is “something that it is like to *be* that organism—something it is like *for* the organism” (Nagel 1974). Ascribing sentience, in this broad sense, to an organism means that the experiences it undergoes will have a certain *subjective character*, but this is not enough to specify how this subjective character might actually *feel*.

In a narrow sense, sentience refers to the capacity to undergo experiences that are felt, specifically, as *attractive* or *aversive*. While narrow sentience requires broad sentience (because experiencing something as attractive or aversive already implies *experiencing* it), it is however possible for an organism to possess phenomenal consciousness (or broad sentience) without having the specific capacity to experience anything as aversive or attractive.

It has been suggested by Godfrey-Smith (Godfrey-Smith 2017) that instead of speaking about narrow and broad sentience, it might be more appropriate to consider different kinds of basic subjectivity. Amongst these, he distinguishes *sensory* subjectivity and *evaluative* subjectivity. Possessing sensory subjectivity implies having the capacity to experience perceptual states, while evaluative subjectivity is equal to what Birch terms ‘narrow sentience’ (Birch 2017), that is, the capacity to experience things as aversive or attractive.

According to Godfrey-Smith, sensory subjectivity and evaluative subjectivity can exist independently from one another and each may have played its own role in evolution. In fact, he lists terrestrial arthropods as a potential example of perceptually complex animals that lack evaluative subjectivity, and gastropods as a possible example of creatures with an evaluative subjectivity and no complex perception (Godfrey-Smith 2017).

What can confidently be said when it comes to the treatment of insects is that it neither matters whether they possess broad sentience, nor whether they possess sensory subjectivity, but whether or not members of these species can *feel pain* or *suffer*, that is, whether they possess *narrow* sentience or *evaluative* sentience. A being that lacks the capacity to experience anything as aversive cannot feel pain and it cannot suffer. Therefore, whatever happens to that being does not matter to that being, and what is done to that being simply does not matter from an ethical perspective. In this respect, we are focusing on aversive experiences because these are the most obviously relevant for ethics, but having the capacity to experience things as attractive, that is, having the capacity to feel pleasure or find certain things enjoyable, also makes a difference to whether or not the way an organism is treated *matters* to that organism.

A handful of ethicists would disagree here, since some ethical theories allow for duties or entitlements to emerge even in the absence of narrow/evaluative sentience (hereafter ‘sentience’). Amongst these we can find, most notably, biocentrists (a position that is reflected in some legislation, see above). For biocentrists, insects would be entitled to moral consideration due the simple fact that they are alive. However, there needs to be a justification as to why being alive entitles one to moral consideration. In the case of sentience, the justification is clear: if an individual does not possess it, then nothing that happens to that being can matter to it.

In the case of life, this justification has proven to be very elusive. Biocentrists often appeal to the notions of teleology (Holm 2017) or welfare (Nolt 2017) in order to justify the moral relevance of life. They argue that all things that are alive are entitled to moral consideration because they have a *good of their own*, which is due to the fact that they have characteristics that are directed towards an end (teleology) or to the fact that things can go better or worse for them (welfare). However, biocentrists are still haunted by the problem of scope. This problem refers to the fact that the definitions of teleology and welfare cannot easily leave out entities such as machines, bacteria, or meteorological phenomena, which also appear to have a good of their own, but whose moral status is dubious at least.

Aside from these problems, the fact remains that the moral relevance of life is disputed by many, while the moral relevance of sentience is very rarely brought into question, with some exceptions (Hsiao 2015). The question of whether insects possess sentience thus seems like a good place to start when determining whether or not they should be granted moral status. Indeed, while sentience is not the only thing that matters when determining the kind of moral consideration that a being is owed, it can plausibly be considered “a threshold condition for membership in the community of beings who have entitlements based on justice” (Nussbaum 2007), i.e. the community of beings who are owed some sort of moral consideration.

### 4.2. Sentience in insects

As opposed to European animal protection law, which (1) at least partly applies to all invertebrates and (2) protects animals not only from the unjustified infliction of negative experiences (like pain and suffering), but also from being harmed, i.e. from negative impacts which are not necessarily accompanied by subjective experiences, in ethics it is a widely accepted claim that sentience is a precondition for moral standing. The problem is that determining whether a certain nonhuman species possesses sentience is very difficult. At the root of this difficulty, we find two classical philosophical problems. The first one is the problem of other minds. This problem emerges because minds are necessarily private. One cannot directly observe another's subjective experiences. One can only observe the behavioral or physiological correlates of these subjective experiences. Thus, we can always doubt whether those around us (even our fellow humans) have minds. Even more important is the existence of another problem: the so-called ‘hard’ problem of consciousness (Chalmers 1995). This boils down to the fact that it is simply not yet known how consciousness emerges from physical structures. Therefore, we cannot yet establish with certainty which neuro-anatomical structures must be present for an individual to be conscious. Thus, we can study the physiology and the behavior of a species in order to attempt to overcome the problem of other minds, but due to the ‘hard’ problem of consciousness, we cannot, as of yet, use the results to conclusively establish whether a certain animal is sentient.

Due to the problem of other minds and the ‘hard’ problem of consciousness, determining whether any nonhuman species is sentient is always a difficult endeavor. But while we can never be absolutely certain that we have found consciousness in another species, we can come pretty close in those cases in which the animals are very similar to humans in neurophysiological and behavioral terms (since, despite the philosophical conundrum involved, no one seriously doubts that other humans have minds).

The more a species differs from us, the more difficulties we will encounter when determining whether its members are sentient. In the case of insects, these difficulties are almost insurmountable. We cannot rely on verbal reports, as in the case of humans, since obviously insects cannot speak. We also cannot rely on arguments from analogy as in the case of vertebrates, since insect physiology differs so much from our own. So, we have to rely on ethological, neurological or neuro-ethological approaches, but these are not as reliable as one would hope. Both the neurological and the neuro-ethological approaches inevitably encounter the ‘hard’ problem of consciousness. Until the latter is solved, we cannot know for sure what the neural correlates of consciousness are. As for ethological approaches, these are also riddled with uncertainty, since the presence of a certain behavior can never guarantee the presence of consciousness. For instance, Adamo argues: “Robots are capable of demonstrating pain-like behavior more similar to our own than any insect … In fact, using behavioral criteria (Sneddon et al. 2014), an argument could be made that some present-day robots are more deserving of ethical treatment and protection (Levy 2009) than are insects” (Adamo 2017). Barron and Klein (Barron and Klein 2016) have also argued that ethological approaches tend to introduce a bias towards animals that do clever or interesting things, even though this may not necessarily be related to consciousness.

There is thus a fundamental epistemological problem: we are, most likely, never going to conclusively determine whether or not insects are sentient. However, it should be noted that there is a very big difference between the size of insect and mammalian brains. The brain of the honeybee, which is very large for an insect, possesses less than one million neurons. In comparison, the brains of mice (6.8 million neurons), rhesus monkeys (6.4 billion neurons) and humans (86 billion neurons) are enormous (Klein and Barron 2016). Some authors have used this difference to argue that insect brains may not be big enough to support sentience (Feinberg and Mallat 2016), while others are confident that the number of neurons is not so important, since the structures relevant for sentience may be implemented on vastly different scales (Merker 2016). It may be the case, however, that insect brains do support sentience but that their experiences are less fine-grained, less complex, than those of animals with much bigger brains, like mammals. This idea is supported by the fact that insects have been shown to continue to use their limbs when they are damaged, eat their own innards and feed while being consumed by another insect (Eisemann et al. 1984). If they do feel pain, these considerations suggest that it may somehow ‘feel less painful’ than it does in other animals. This may be a relevant consideration to take into account when their interests conflict with those of more complex animals.

### 4.3. Appealing to a precautionary principle

As discussed before, it is virtually impossible for us to obtain conclusive evidence of insect sentience. However, as is often remarked in comparative cognition, absence of evidence is not equal to evidence of absence. So, the fact that there are severe epistemological obstacles to the study of insect sentience does not, in and of itself, mean that insects are not sentient. In fact, it may very well be that insects can feel pain and suffer and we simply lack the tools to establish it scientifically. If insects were indeed sentient, we might inadvertently be causing them serious suffering with our actions. Waiting until we have strong evidence of their capacity to undergo conscious experiences might take too long, and thus a better strategy might be to treat them *as if* we already knew that they are sentient. Several ethicists have defended this strategy by suggesting that we follow some form of precautionary principle.

It would be absurd to include within the protection of a precautionary principle every single being for whom we don't have conclusive evidence of their lack of sentience, since this would even require taking measures to protect inanimate objects from pain, in case panpsychism were to be true. Therefore, any precautionary principle needs to be narrowed down. Along these lines, Birch (Birch 2017) has formulated a precautionary principle that commits us to include within animal protection legislation all animals for which there is statistically significant evidence of the presence of at least one credible indicator of sentience in at least *one species of the order* that those animals belong to. The sorts of behaviors that he considers to be credible indicators of sentience are things like self-delivering analgesics, weighing the preference to avoid a noxious stimulus against other preferences, and demonstrating an avoidance of locations where noxious stimuli have been found before.

Other authors believe we should be less cautious towards animals whose moral status is as uncertain as insects and reserve greater protection for those species whose sentience has been well established scientifically. Fischer, for instance, defends a precautionary principle that establishes that we should treat animals that are probably conscious as though they were definitely conscious only in those cases where it wouldn't prevent us from fulfilling any obligation towards a being that is definitely conscious (Fischer 2016). As for beings who are perhaps conscious (a group where Fischer locates insects), we should treat them as definitely conscious only when their interests don't conflict with beings that are definitely or probably conscious.

Because prescribing a specific course of action can be very problematic, due to the high number of intervening factors, potential consequences and other relevant considerations that must be taken into account, some ethicists consider that we should be even less restrictive in our prescriptions. Knutsson & Munthe (Knutsson and Munthe 2017), for example, take a virtue ethics approach and defend the need to cultivate “…a character trait of being disposed to consider the possible moral importance of these beings”. This way, rather than establishing that we should always treat these beings as though they were sentient, we should be open to taking these precautions or not, depending on the situation, the potential consequences, the price to pay, and so on. While this sort of precautionary approach allows us to easily maneuver in the case of a conflict of interests or a situation with high uncertainty regarding the causal implications of our actions, one could also argue that it is excessively vague and permissive, and that it could give too much room for a mistreatment of possibly sentient beings.

### 4.4. Treatment of insects if they are sentient

If we were to establish that a certain species of insect that is currently raised for food is sentient (something that, as we have seen, is not at all easy to determine), the immediate question arises: what should we do about it? One possibility would be to conclude that members of this species should be granted full moral status, which implies a moral right to freedom and to life, such as is defended by many animal rights activists and theorists with respect to other species that are traditionally raised for food. This would imply that we are no longer morally allowed to use this species of insect in our food production.

This conclusion, however, immediately leads to several problems. Firstly, it seems that other farmed species, such as pigs, have a more urgent and justified claim to being liberated, due to their much more complex ethological needs, which arguably cannot be catered for in modern husbandry systems. As Fischer has argued (Fischer 2016), we may have underestimated the minds of insects, but it is highly unlikely that they will rival the minds of highly complex animals like pigs. As Nussbaum notes (Nussbaum 2004), the ways in which a being can be harmed depend on the complexity of that being. More complex beings will be capable of suffering more types of harm than less complex beings. If being raised for food means suffering more types of harm for pigs than it does for insects, then arguably the former have a stronger case for being liberated.

Moreover, a plausible case can be made to argue that those who defend farm animal liberation in fact have a moral obligation to eat insects. This idea has been defended by Fischer (Fischer 2016a) and it is based on the fact that the techniques used for planting and harvesting fruits and vegetables routinely harm or kill animals that happen to be in the field and that arguably have a higher claim to moral consideration than insects, such as mice or rabbits. Combining a vegan diet with the occasional consumption of insects might actually result in less harm being delivered to animals with complex subjective experiences than a strictly vegan diet. This argument might not work, though, if the idea that raising insects for food implies an instrumentalisation or a commodification that is not present when animals are killed as side-effects of the use of agricultural machinery is factored in. However, it is unclear whether this notion of instrumentalisation or commodification can be easily applied to the case of insects, and whether it would be enough to trump the interest of mice and rabbits in continuing to live.

Fischer further notes that ascribing full moral status to insects may also have many undesirable consequences (Fischer 2016): a huge number of them are killed by cars—would we then have a moral obligation not to engage in non-essential driving? Or should we let fleas take over our dog's body? And what should we do about insecticides? Ascribing full moral status to insects would imply a radical change in our lifestyles. This is not enough to conclude that insects have no moral status (slave-owners also had to face a radical change in their lifestyles when slavery was abolished), but together with the other considerations it suggests that, with respect to insects, their possession of sentience would likely point us in the direction of *welfare measures*, rather than outright liberation.

A study by Adamkova et al. suggests that taking the welfare of edible insects into account may actually have positive consequences that go beyond insect welfare (Adámková et al. 2017). In the case of mealworms, the species studied, they established that subjecting the mealworms to nutritional deprivation affected their welfare, but also had a negative effect on their nutritional value and the economic aspects of their breeding. They also found that the moment of death as well as the way of killing influenced the nutritional value and the quality of the meat obtained. Death by freezing, as opposed to boiling, was found to be better from both a welfare and a nutritional perspective. Thus, in addition to the ethical advantages, there may also be pragmatic reasons to ensure the welfare of insects raised for food.

## 5. Knowledge gaps and future steps

Following Knutsson & Munthe (Knutsson and Munthe 2017) we can identify six challenges that need to be overcome in the field of insect ethics. The first challenge is to determine whether or not insects are sentient. It is clear from what was discussed above how difficult answering this question is going to be. The second challenge is to determine to what degree they are sentient, meaning whether their sentience is of the morally relevant sort and whether their experiences are as fine-grained as those of other animals.

The third challenge is to establish how many beings are involved in insect rearing and whether these numbers count morally. While we have already seen that there are both environmental and ethical advantages to consuming insects, the fact is that many more individuals will have to die if we want to obtain the same nutritional yield that we get from consuming traditional sources of meat. We have to reach a consensus on whether we consider these numbers to count morally. While from the perspective of most consequentialist ethics these numbers would count, there have been some ethicists who have argued that numbers are irrelevant for morality, i.e. that it is just as bad to kill one individual as it is to kill a thousand (Taurek 1977).

The three final challenges that Knutsson & Munthe identify relate to the costs that may come from ascribing moral status to insects (Knutsson and Munthe 2017). The first one is the problem of how to assess the complex causal effects of our actions. For instance, if we were to assign moral status to insects and stop using pesticides, this might have long-term effects on the population of insects and other animals, which could lead to more suffering on the whole than using pesticides. The second of these final challenges is how to balance different interests and values. It is clearly inevitable that the interests of different species will conflict, so there needs to be a way of establishing whose interests and which values will be favored. And, lastly, it needs to be acknowledged that precaution does not come for free, so the final challenge is to determine what price we are willing to pay in exchange for behaving cautiously towards beings with uncertain sentience.

Aside from these challenges, we should obviously attempt to provide welfare measures for the insects reared for food, not only because of the ethical advantages, but also for pragmatic reasons, as discussed above. The biggest knowledge gap that we have to overcome is the topic of insect welfare, which is so understudied that searching for “insect welfare” on Google Scholar delivers: “Did you mean insect warfare?”. In addition, an important point to bear in mind is that “…insect biodiversity is too large to generalize upon welfare standards” (De Goede, Kapsomenou, and Peters 2013). So it is not enough to study a couple of species and then develop welfare standards just based on these results. A good deal of research effort should therefore go to studying the welfare of the insects currently reared for food and feed, and determining what the costs of implementing welfare standards would be.

## 6. Conclusion

The increasingly propagated usage of farmed insects for human nutrition raises issues regarding food safety, consumer information and animal protection. While human-related challenges have been addressed by supranational law since the late 1990s, animal-related problems brought about by large-scale farming of insects have been neglected so far. National legislation differs with regard to the protection of insects, but generally fails to address farming and killing of insects comprehensively.

To eliminate this deficiency it would be necessary to fully include insects into the scope of animal protection legislation and to define standard requirements for the rearing of the most commonly farmed insects but also for insect taxa bred as feed and kept as pets. On the other hand there is a great need for research in the area of insect welfare, especially regarding species-specific needs, health, farming systems and humane methods of killing. From an animal protection point of view these issues should be investigated and satisfyingly solved before propagating and establishing intensive husbandry systems for insects as a new type of mini-livestock factory farming.

## 7. Summary

While seeking novel food sources to feed the increasing population of the globe, several alternatives have been discussed, including algae, fungi or *in vitro* meat. The increasingly propagated usage of farmed insects for human nutrition raises issues regarding food safety, consumer information and animal protection. In line with law, insects like any other animals must not be reared or manipulated in a way that inflicts unnecessary pain, distress or harm on them.

Currently, there is a great need for research in the area of insect welfare, especially regarding species-specific needs, health, farming systems and humane methods of killing. Recent results from neurophysiological, neuroanatomical and behavioral sciences prompt caution when denying consciousness and therefore the likelihood of presence of pain and suffering or something closely related to it to insects.

From an animal protection point of view, these issues should be satisfyingly solved before propagating and establishing intensive husbandry systems for insects as a new type of mini-livestock factory farming.
